# P17, an Original Host Defense Peptide from Ant Venom, Promotes Antifungal Activities of Macrophages through the Induction of C-Type Lectin Receptors Dependent on LTB4-Mediated PPARγ Activation

**DOI:** 10.3389/fimmu.2017.01650

**Published:** 2017-11-30

**Authors:** Khaddouj Benmoussa, Hélène Authier, Mélissa Prat, Mohammad AlaEddine, Lise Lefèvre, Mouna Chirine Rahabi, José Bernad, Agnès Aubouy, Elsa Bonnafé, Jérome Leprince, Bernard Pipy, Michel Treilhou, Agnès Coste

**Affiliations:** ^1^UMR 152 Pharma Dev, Université de Toulouse, IRD, UPS, Toulouse, France; ^2^IRD, UMR 152, Toulouse, France; ^3^EA7417 BTSB, Université Fédérale Toulouse Midi-Pyrénées, INU Champollion, Albi, France; ^4^INSERM U982, PRIMACEN, IRIB, Université de Rouen, Mont-Saint-Aignan, France

**Keywords:** Macrophages, host defense peptide, antimicrobial peptides, *Candida albicans*, C-type lectin receptors, arachidonic acid metabolism, PPARγ, inflammasome

## Abstract

Despite the growing knowledge with regard to the immunomodulatory properties of host defense peptides, their impact on macrophage differentiation and on its associated microbicidal functions is still poorly understood. Here, we demonstrated that the P17, a new cationic antimicrobial peptide from ant venom, induces an alternative phenotype of human monocyte-derived macrophages (h-MDMs). This phenotype is characterized by a C-type lectin receptors (CLRs) signature composed of mannose receptor (MR) and Dectin-1 expression. Concomitantly, this activation is associated to an inflammatory profile characterized by reactive oxygen species (ROS), interleukin (IL)-1β, and TNF-α release. P17-activated h-MDMs exhibit an improved capacity to recognize and to engulf *Candida albicans* through the overexpression both of MR and Dectin-1. This upregulation requires arachidonic acid (AA) mobilization and the activation of peroxisome proliferator-activated receptor gamma (PPARγ) nuclear receptor through the leukotriene B4 (LTB4) production. AA/LTB4/PPARγ/Dectin-1-MR signaling pathway is crucial for P17-mediated anti-fungal activity of h-MDMs, as indicated by the fact that the activation of this axis by P17 triggered ROS production and inflammasome-dependent IL-1β release. Moreover, we showed that the increased anti-fungal immune response of h-MDMs by P17 was dependent on intracellular calcium mobilization triggered by the interaction of P17 with pertussis toxin-sensitive G-protein-coupled receptors on h-MDMs. Finally, we also demonstrated that P17-treated mice infected with *C. albicans* develop less severe gastrointestinal infection related to a higher efficiency of their macrophages to engulf *Candida*, to produce ROS and IL-1β and to kill the yeasts. Altogether, these results identify P17 as an original activator of the fungicidal response of macrophages that acts upstream PPARγ/CLRs axis and offer new immunomodulatory therapeutic perspectives in the field of infectious diseases.

## Introduction

Antimicrobial peptides (AMPs), also called host defense peptides (HDPs), are small molecules produced by all living forms including bacteria, insects, plants and vertebrates. These peptides are especially found in skin and intestine; thus, they constitute the first line of organism immune defense (1). Although HDPs can be cationic or anionic, most of them are cationic molecules with an amphipatic structure (2).

Unlike antibiotics, cationic HDPs have a microbicidal activity against a broad spectrum of pathogens including bacteria, yeast, and viruses. They exercise their direct antimicrobial effect essentially by interacting with negatively charged membranes of target cells inducing membrane destabilization and cell death (1, 3, 4). These HDPs can also inhibit intracellular pathways critical for pathogen survival (5–7). Beside their microbicidal activity, most of cationic HDPs exhibit modulatory functions on immune cells. HDPs are produced by and act on several cell types including innate and adaptive immune cells (8–10). Their functions are multiples and depend on their structure, the microenvironment, and the target cell. In humans, the two main classes of HDPs are cathelicidins and defensins (11, 12). LL-37, the only member of the human cathelicidin AMP family, is well known to modulate innate and adaptive immune responses. Regarding adaptive immune response, this peptide induces T lymphocytes chemotaxis and regulates the activation of antigen-presenting cells and Th1 polarization of T cells (13). This AMP can also contribute to innate immune response by controlling the activation of monocytes, macrophages, and dendritic cells (13). Indeed, it was previously described that LL-37 regulates cytokine and chemokine genes expression and protein secretion in human monocytes and macrophages (14–16). Moreover, this AMP promotes directly microbicidal activities of monocytes, macrophages, and neutrophils by increasing pathogen phagocytosis and reactive oxygen species (ROS) release (14, 15, 17–19). This human cathelicidin is also involved in immune cells apoptosis, angiogenesis, and wound-healing regulation (20–24). Similar to LL-37, immunomodulatory effects on innate immunity were also described for defensins and several HDPs found in insect venom (25, 26). Usually, their effects on immune cells are mediated through G-protein-coupled receptors (GPCRs). Among the HDP-activated GPCRs, the *N*-formylmethionine-leucyl-phenylalanine receptors 1 and 2 (FPR1 and FPR2) and the chemokine receptors are the most involved (27–30).

Our laboratory has previously isolated two original HDPs from ant venom of *Tetramorium bicarinatum* (31). These peptides, named P16 (Bicarinalin) and P17, are cationic, C-terminal amidated, and adopt an α-helix conformation. In this previous study, the authors demonstrated a potent and broad antibacterial activity for the Bicarinalin (31). Although no microbicidal activity for P17 was demonstrated, this HDP could be a good candidate to modulate immune response because of its structural properties and its low toxicity on human cells.

Despite the growing knowledge with regard to the immunomodulatory properties of HDPs, little is known about how they control macrophage differentiation and its associated microbicidal functions. Emerging evidence indicates that the state of macrophage polarization plays a critical role in the host susceptibility against infections. The following two programs broadly classify polarized macrophages: classical (M1) and alternative (M2) (32, 33). The M1 program arises from type 1 inflammatory conditions (e.g., IFNγ) and is characterized by elevated levels of opsonic receptors. M1 macrophages highly produce pro-inflammatory effector molecules, such as reactive oxygen and nitrogen species, and pro-inflammatory cytokines [interleukin (IL)-1β, TNF-α, IL-6, and IL-12]. These macrophages contribute to inflammation and microbial killing. M2 alternative macrophages are characterized by abundant levels of the anti-inflammatory cytokine IL-10 and non-opsonic receptors, such as C-type lectin receptors (CLRs) and scavenger receptors. These alternative-activated macrophages can also efficiently participate to pathogen clearance through CLR-mediated recognition and phagocytosis (34–36).

Among the CLRs, Dectin-1 and mannose receptor (MR) of the phagocytic system have been described to be essential in anti-fungal functions of macrophages (34, 35, 37–42).

After binding of β-glucans and mannans, the major cell wall components of *Candida albicans*, these CLRs act in a cooperative manner to activate syk-p47^phox^ axis essential to ROS production and inflammasome-dependent signaling pathways for IL-1β release (35, 36, 43). Thus, the induction of these two CLRs at the surface of macrophages is critical for the fungicidal response.

The balance of macrophage differentiation toward an alternative phenotype, known to highly express MR and Dectin-1, is controlled by the nuclear receptor peroxisome proliferator-activated receptor gamma (PPARγ) activation (34, 35, 43). Among PPARγ ligands, lipids from the metabolism of arachidonic acid (AA) through the COX-1/COX-2 cyclooxygenases and 5- and 12/15-lipoxygenases (LOX), are considered to be critical for PPARγ endogenous activation (37, 44, 45). We have previously demonstrated that PPARγ activation in macrophages promotes the transcription of Dectin-1 and MR and its associated anti-fungal functions (34, 43, 46). Although the processes leading to PPARγ activation, such as AA release and its subsequent metabolic conversion, are important aspects of macrophage alternative polarization and CLR-dependent anti-fungal defense, the impact of HDPs on this signaling is not yet elucidated.

In the present study, we demonstrated that P17 promotes alternative activation of the human monocyte-derived macrophages (h-MDMs) characterized simultaneously by a CLRs signature composed of MR and Dectin-1 and a pro-inflammatory profile. Interestingly, this HDP also improves the recognition and the phagocytosis of *C. albicans* by h-MDMs through the overexpression of MR and Dectin-1. This upregulation requires AA release and the activation of PPARγ through the leukotriene B4 (LTB4) production. The activation of AA/LTB4/PPARγ/Dectin-1-MR axis by P17 triggers ROS production and inflammasome-dependent IL-1β release critical in the fungicidal activity of P17-activated h-MDMs. Finally, we validated the efficiency of P17 to eliminate *C. albicans in vivo*. Indeed, P17-treated mice infected with *C. albicans* develop less severe gastrointestinal infection related to a higher ability of their macrophages to engulf *Candida*, to produce ROS, IL-1β, and to kill *C. albicans* as compared to untreated mice. Altogether, these results identify P17 as an original activator of the fungicidal response of macrophages and support that this novel HDP may constitute promising compound to restrain fungal infections.

## Materials and Methods

### P17 AMP

The sequence of the P17 peptide (LFKEILEKIKAKL-NH2) was characterized by *de novo* sequencing using mass spectrometry and Edman degradation (31). P17 peptide and its randomly designed C-terminal amidated scrambled counterpart (KIKEEKFLLKLI-NH2) were synthesized on a Liberty microwave assisted automated peptide synthesizer (CEM, Saclay, France) at a purity grade higher than 99% as previously described (31, 47). The authenticity and the molecular identity of the synthetic peptides were controlled by MALDI-TOF-MS.

### Preparation of h-MDMs

Human peripheral blood mononuclear cells were isolated from the blood of healthy volunteers by a density gradient centrifugation method on Lymphoprep (Abcys). Monocytes were isolated from mononuclear cells by adherence to plastic for 2 h in special macrophage serum-free medium (SFM; Life Technologies) with l-glutamine at 37°C in a humidified atmosphere containing 5% CO_2_. Non-adherent cells were removed by washing with Hanks’ balanced salt solution (HBSS) (Gibco, Invitrogen), and the remaining adherent cells (>85% monocytes) were incubated in SFM medium. The h-MDMs were obtained after 24 h of culture in SFM medium. Adherent h-MDMs were pre-incubated 30 min or not before the addition of P17 (200 µg/ml) with GW9662 (1 nM; Santa Cruz Biotechnology), MAFP (20 µM; Calbiochem), MK-886 (10 µM; Calbiochem), *N*-acetyl-cysteine (10 mM; Sigma), or Z-Vad-FMK (ZVAD) (50 µM; Calbiochem) for 30 min.

### *C. albicans* Strains

The strain of *C. albicans* used throughout these experiments was isolated from a blood culture of a patient in the Toulouse-Rangueil University Hospital (98/26135). The isolate was identified as *C. albicans* based on common laboratory criteria and cultured on Sabouraud dextrose agar (SDA; Biorad, Hercules, CA, USA) plates containing gentamicin and chloramphenicol. *C. albicans* was maintained by transfers on SDA plates. Growth from an 18- to 24-h SDA culture of *C. albicans* was suspended in sterile saline buffer (HBSS; Life Technologies). In all experiments, the h-MDMs were challenged with blastospores.

### Killing Assay

Human monocyte-derived macrophages were treated with P17 (200 µg/ml) for 24 h and were allowed to interact for 40 min at 37°C with *C. albicans* blastospores (at a ratio of 0.3 yeast per macrophage) as previously described (36, 40). Unbound yeasts were removed by four washes with medium. h-MDMs were then incubated at 37°C for 4 h. After incubation, the medium was removed and cells were lysed. The CFU of *C. albicans* were quantified after plating on Sabouraud plates for 24–48 h at 37°C.

In some experiments, monocyte-derived macrophages were incubated with 1 µg per well of MR siRNA (Santa Cruz Biotechnology sc-45360) and/or Dectin-1 siRNA (Santa Cruz Biotechnology sc-63276) for 6 h into siRNA transfection medium (siRNA reagent system, Santa Cruz Biotechnology sc-45064) according to the manufacturer’s instructions before the addition of P17.

### Binding and Phagocytosis Assay

Human monocyte-derived macrophages were treated with P17 (200 µg/ml) for 24 h and were then challenged with GFP-labeled yeasts at a ratio of 6 blastospores per macrophage. In some experiments, the monocyte-derived macrophages were incubated at 4°C for 20 min with 500 µg/ml of mannans and/or laminarins before the addition of the yeasts. The binding was performed at 4°C and the phagocytosis was initiated at 37°C. The number of *C. albicans* bound or engulfed by macrophages was determined by fluorescence quantification using the Envision fluorimetry-based approach (Perkin Elmer).

### Assay for ROS Production

Human monocyte-derived macrophages were treated with P17 (200 µg/ml) for 24 h and ROS production was measured by chemiluminescence in the presence of 5-amino-2,3-dihydro-1,4-phthalazinedione (luminol; Sigma) using a thermostatically (37°C) controlled luminometer (Envision; Perkin Elmer). The generation of chemiluminescence was monitored continuously for 1 h and 30 min after challenge or not with *C. albicans* blastospores (yeast-to-macrophage ratio: 3:1). Statistical analysis was performed using the area under the curve expressed in counts × seconds.

### ELISA Cytokine Titration

Human monocyte-derived macrophages were treated with P17 (200 µg/ml) for 24 h and challenged with *C. albicans* blastospores at a ratio of 3 yeasts per macrophage for 8 h. The release of TNF-α, IL-1β, IL-12, and IL-10 in the cell supernatants was determined with a commercially available OptiEIA kit (BD Biosciences) according to the manufacturer’s instructions.

### Western Blot Analysis

Human monocyte-derived macrophages were treated with P17 (200 µg/ml) for 24 h and challenged for 8 h with *C. albicans* blastospores at a ratio of 3 yeasts per macrophage. h-MDMs were lysed with RIPA buffer (Sigma) and protein extracts were separated in SDS-PAGE as previously described (37). After protein transfer, membranes were incubated overnight at 4°C with a rabbit anti-phosphorylated p47^phox^ (Assay biotechnology, A1171; 1/260), a rabbit anti-caspase-1 (Biovision, 3019-100; 1/100), or a goat anti-GAPDH antibody (Cell Signaling, #5174; 1/1,000) and then for 1 h at room temperature with a peroxidase conjugated secondary antibody. Membranes were washed, and proteins of interest were visualized with WesternBright™ ECL (Advansta) or the SuperSignal West Pico Chemiluminescent Substrate (ThermoScientific) for the GAPDH. Images have been cropped for presentation.

### Flow Cytometry

Human monocyte-derived macrophages were treated with P17 (200 µg/ml) for 24 h and then incubated with ice-cold PBS and gently scraped. Collected cells were centrifuged at 1,500 rpm for 10 min, and the cell pellet was suspended in PBS medium supplemented with 1% fetal calf serum (FCS). Surface expressed MR, Dectin-1, DC-SIGN, CD16, or CD36 was detected, respectively, using PerCP-Dectin-1 monoclonal antibody (mAb; R&D FAB1859C-100, 1/40), PE-DC-SIGN (mAb; BD Biosciences 551 265, 1/20), FITC-CD16 (mAb; PNIM 0814, 1/20), or APC-CD36 monoclonal antibodies (mAb; BD Biociences 550 956, 1/40). To evaluate the MR surface expression, we have used MR-specific ligand conjugated with FITC (Sigma A7790, 1 mg/ml, 1/100). All staining were performed on PBS^−/−^ 1% FCS medium. A population of 10,000 cells was analyzed for each data point. All analyses were carried out in a Becton Dickinson FACScalibur using the FACSDiva version 6.2 software.

### Reverse Transcription and Real-time PCR

Human monocyte-derived macrophages were treated with P17 (200 µg/ml) and/or LTB4 (100 nM; Cayman Chemical Company) for 8 h.

The mRNA preparation was made using the EZ-10 Spin Column Total RNA Minipreps Super Kit (Bio Basic) using the manufacturer’s protocol. Synthesis of cDNA was performed according to the manufacturer’s recommendations (Thermo electron). RT-qPCR was performed on a LightCycler 480 system using LightCycler SYBR Green I Master (Roche Diagnostics). The primers (Eurogentec) were designed with the software Primer 3. GAPDH mRNA was used as the invariant control. Serially diluted samples of pooled cDNA were used as external standards in each run for the quantification. Primer sequences are listed in Table 1.

**Table 1:** Human primer sequences used in qPCR analysis.

Gene	5′–3′ sequence	
*Alox5*	Antisense	ACT-GGA-AAC-ACG-GCA-AAA-AC
	Sense	TTT-CTC-AAA-GTC-GGC-GAA-GT
*Itgam* (CD11b)	Antisense	TTG-CAT-CCA-TCT-CAA-ATC-CA
	Sense	CTC-CCA-AAG-TGC-TGG-GAT-TA
*Fcgr3* (CD16)	Antisense	TAC-AGC-GTG-CTT-GAG-AAG-GA
	Sense	GCA-CCT-GTA-CTC-TCC-ACT-GT
*Fcgr2* (CD32)	Antisense	CCA-AAG-GCT-GTG-CTG-AAA-CT
	Sense	TAC-TCC-CCG-CTG-TCA-TTG-TT
*Cd36*	Antisense	TGA-TAG-GTG-CAG-CAA-AGC-AC
	Sense	TGT-AAC-CCA-GGA-CGC-TGA-GG
*Clec7a* (Dectin-1)	Antisense	CCA-AGC-ATA-GGA-TTC-CCA-AAA
	Sense	AAA-AGG-ATC-GTG-TGC-TGC-ATC
*Ptgs2* (COX-2)	Antisense	TGA-GCA-TCT-ACG-GTT-TGC-TG
	Sense	TGC-TTG-TCT-GGA-ACA-ACT-GC
*Pla2g4a* (cPLA2)	Antisense	GCC-TTG-GTG-AGT-GAT-TCA-GCT
	Sense	AGA-TTC-AAG-CCC-AGC-ATG-AAG
*Cd209* (DC-SIGN)	Antisense	GGG-CAT-GGA-GGC-TCC-AC
	Sense	CAA-CTT-AGA-AAC-AGC-CAA-ATG-GAA
*Alox5ap* (FLAP)	Antisense	ACC-CGC-TCA-AAG-GCA-ATG-G
	Sense	CAC-GAA-AGC-AGG-ACC-CAG-A
*Gapdh*	Antisense	AGG-TCG-GAG-TCA-ACG-GAT-TT
	Sense	ATC-TCG-CTC-CTG-GAA-GAT-GG
*Il1b*	Antisense	CAG-CCA-ATC-TTC-ATT-GCT-CA
	Sense	AGG-CAG-AGA-GGG-AAG-GAG-AG
*Il6*	Antisense	TAC-CCC-CAG-GAG-AAG-ATT-GT
	Sense	TTT-TCT-GCC-AGT-GCC-TCT-TT
*Il12*	Antisense	TGG-GTG-GGT-CAG-GTT-TGA-TG
	Sense	GCC-CAG-CTG-CTG-AGG-AGA-GT
*Il10*	Antisense	TGC-AAA-ACC-AAA-CCA-CAA-GA
	Sense	TCT-CGG-AGA-TCT-CGA-AGC-AT
*Il1ra*	Antisense	TGG-GAA-TCT-CAG-ATG-GGA-AG
	Sense	CTG-TGT-CCC-CCA-GAA-CTT-GT
*Tgfb1*	Antisense	ACT-GAG-GGG-AAG-GGA-CAA-CT
	Sense	TCG-GTA-CCA-GGT-GAG-GGT-AG
*p47^phox^*	Antisense	CCT-CAT-TGT-CCA-GTG-TGG-TG
	Sense	TCT-TCC-GTC-TCG-TCA-GGA-CT
*Lta4h*	Antisense	ACT-GCT-TGG-AGG-ACC-AGA-GA
	Sense	GGA-AAG-CAT-TAG-CAG-GCA-AG
*Mrc1* (MR)	Antisense	GGC-GGT-GAC-CTC-ACA-AGT-AT
	Sense	ACG-AAG-CCA-TTT-GGT-AAA-CG
*Ptges* (PGES)	Antisense	CAT-GTG-AGT-CCC-TGT-GAT-GG
	Sense	GAC-TGC-AGC-AAA-GAC-ATC-CA
*Pparg*	Antisense	GCT-GTG-CAG-GAG-ATC-ACA-GA
	Sense	GGG-CTC-CAT-AAA-GTC-ACC-AA
*Tnfα*	Antisense	TCC-TTC-AGA-CAC-CCT-CAA-CC
	Sense	AGG-CCC-CAG-TTT-GAA-TTC-TT

### AA Mobilization and EIA Lipid Quantification

Human monocyte-derived macrophages were pre-labeled with [^3^H]AA. Briefly, h-MDMs (5 × 10^5^ per well in 48-well plates) were cultured for 18 h at 37°C in the presence of P17 (200 µg/ml) and 1 μCi/ml [^3^H]AA. The culture medium was then removed and pre-labeled macrophages were washed three times with 0.5 ml SFM. The cells were challenged with *C. albicans* blastospores at a ratio of 3 yeasts per macrophage for 2 h. The [^3^H]AA metabolites released into the culture medium were quantified by measurement of the radioactivity by beta liquid scintillation counting using a 1217 Wallac Rackbeta LKB 1217, as previously described (37).

### Determination of Intracellular Calcium Concentration

Intracellular calcium concentration was measured using the fluorescent probe Fluo 3-AM (Molecular Probe). Briefly, h-MDMs (1.5 × 10^5^) were incubated with 11.5 × 10^−6^ M Fluo 3-AM for 30 min at 37°C. The time course of the intracytosolic Ca^2+^ level was recorded every 0.5 s for a total period of 3 min after the addition of P17 (200 µg/ml). In desensitization experiments, a second injection of bacterial *N*-formylmethionine-leucyl-phenylalanine peptide (fMLP) or P17 was performed at the end of fluorescence record and the time course of the intracytosolic Ca^2+^ level was recorded for supplemental 3 min. In some experiments, h-MDMs were pre-incubated with U73122 (2 µM) or with calcium-free HBSS for 10 min before the addition of P17. Fluorescence was quantified using the Envision fluorimetry-based approach (Perkin Elmer).

### *In Vivo* Experiment

Male mice aged 12 weeks on C57BL/6 background were used for *in vivo* experiments. Mice were bred and handled by the protocols approved by the Conseil Scientifique du Centre de Formation et de Recherche Experimental Medico Chirurgical and the ethics board of the Midi-Pyrénées ethic committee for animal experimentation (Approval no B3155503). All cages were changed twice weekly, and all manipulations of the animals were carried out in a laminal blow hood under aseptic conditions. The photoperiod was adjusted to 12 h light and 12 h dark. C57BL/6 mice were purchased from Janvier (France).

The gastrointestinal infection (GI) with the *C. albicans* was established by oral infection with 50 × 10^6^ *C. albicans* per mouse. To establish esophageal and GI candidiasis in mice, we performed intra-esophageal infection with 5 × 10^7^ viable cells of *C. albicans* in sterile saline solution. No antibiotic or immunosuppressive treatment was used to facilitate mucosal infection by *C. albicans* of the oral cavity and GI tract.

Mice were treated intraperitoneally with P17 (10 µg per mouse) 1 day before the day of the infection with *C. albicans* and then every 2 days (four injections). Control groups received saline solution. Therapeutic studies were performed on separate groups of six mice infected with *C. albicans* treated or not with P17.

The body weight of each mouse was recorded daily, and the condition of each mouse was assessed twice daily. Feces were collected on days 4 and 5 post-infections. At day 6 post-infection, all mice were euthanized using CO_2_ asphyxia and the cecum and colon were aseptically removed to evaluate *C. albicans* colonization. To quantify the number of viable *C. albicans* in cecum, colon, and feces, each tissue sample removed was mechanically homogenized in 1 ml of saline with 100 U of penicillin/ml and 100 µg of streptomycin/ml. Serial dilutions of homogenate were plated onto SDA for quantitative determination of the number of *C. albicans* in the tissue samples. Plates were incubated at 37°C for 1–2 days and the number of colonies was counted.

After euthanasia, resident peritoneal cells were harvested by washing the peritoneal cavity with 5 ml of sterile 199 medium with Hanks’ salts as previously described (6). Collected cells were centrifuged at 400 × *g* for 8 min, and the cell pellet was suspended in SFM optimized for macrophage culture (Invitrogen Life Technologies). Cells were allowed to adhere for 2 h at 37 C and 5% CO_2_. Non-adherent cells were then removed by washing with HBSS. The macrophage monolayers were used to investigate their capacity to bind, to engulf and to kill *C. albicans*, and to produce ROS and IL-1β in response to yeast challenge.

### Statistical Analysis

For each experiment, the data were subjected to one-way analysis of variance followed by the means multiple comparison method of Bonferroni–Dunnett. *p* < 0.05 was considered as the level of statistical significance.

## Results

### P17 Promotes Alternative Activation of h-MDMs Characterized by an Inflammatory Signature

**Figure 1:**
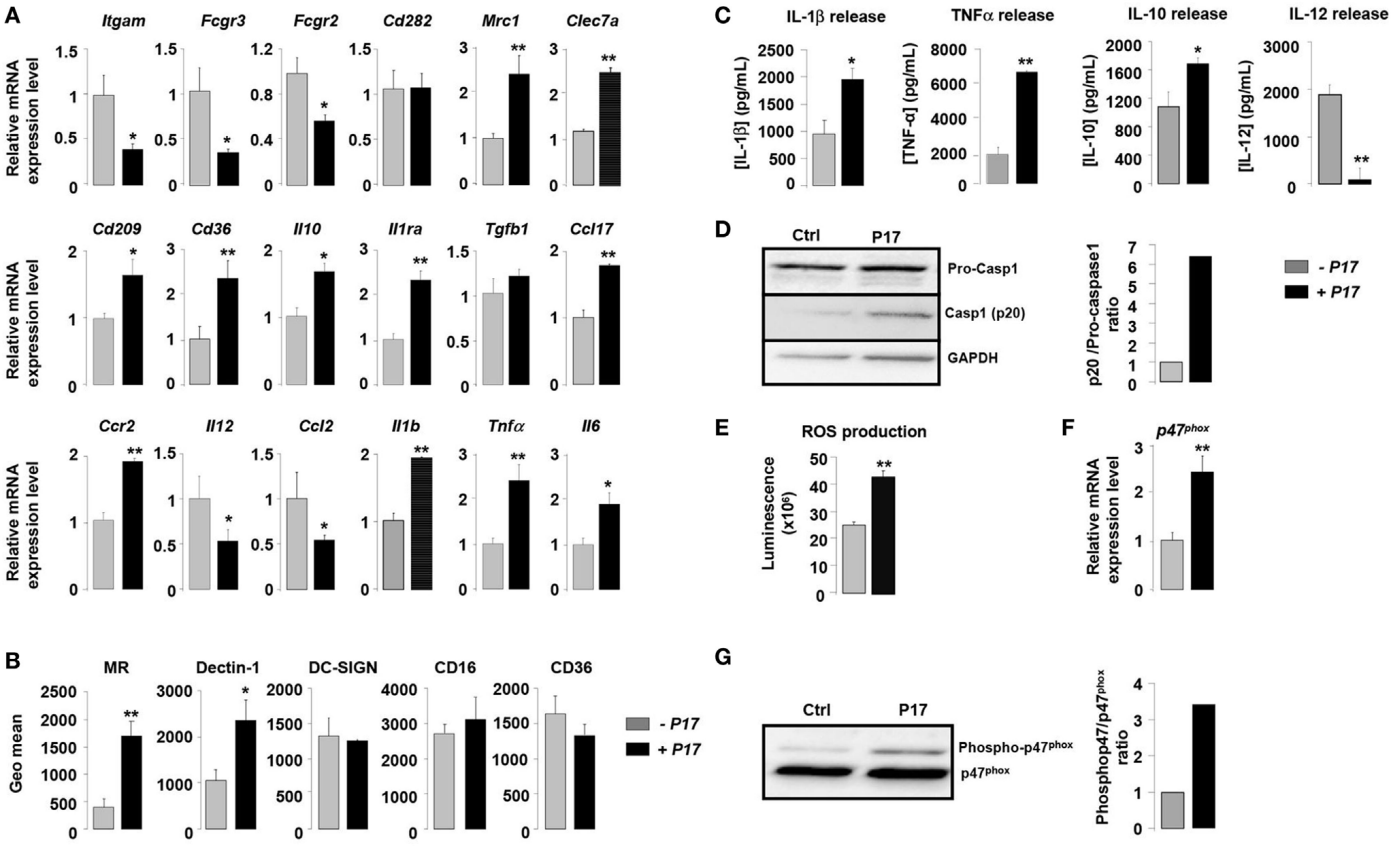
P17 mediates an alternative phenotype of human monocyte-derived macrophages (h-MDMs) characterized by C-type lectin receptor (CLR) and inflammatory signatures. **(A)** Gene expression analysis of markers of classical and alternative polarization in h-MDMs treated or not with P17. The results were represented in fold induction relative to the untreated h-MDMs control. **(B)** Mannose receptor (MR), Dectin-1, DC-SIGN, CD16, and CD36 protein expressions in h-MDMs treated or not with P17. Graphs represent geomean fluorescence quantification for the indicated proteins. **(C)** TNF-α, interleukin (IL)-1β, IL-12, and IL-10 release by h-MDMs treated or not with P17 after challenge with *Candida albicans*. **(D)** Immunoblot analysis of caspase-1 p20 fragment cleavage in h-MDMs treated or not with P17 after *C. albicans* challenge. Band intensity was quantified using the ImageJ software and was represented as the ratio between the band intensities of p20 and of pro-caspase-1. **(E)** Reactive oxygen species production by h-MDMs treated or not with P17 after challenge with *C. albicans*. **(F)** Gene expression of P47^phox^ in h-MDMs treated or not with P17. The results were represented in fold induction relative to the untreated h-MDMs control. **(G)** Phosphorylated p47^phox^ immunoblot after *C. albicans* challenge in h-MDMs treated or not with P17. Band intensity was quantified using the ImageJ software and was represented as the ratio between the band intensities of phosphorylated p47^phox^ and of P47^phox^. Results correspond to mean ± SEM of triplicates. Data are representative of three independent experiments. *p < 0.05, **p < 0.01 compared to the respective untreated control.

In order to assess the impact of P17 in h-MDMs differentiation, we evaluated the expression of specific markers of classical and alternative activation in P17-treated h-MDMs. Overall, P17-treated h-MDMs displayed a downregulation of membrane receptors characteristics of classical M1 polarization, such as CD11b (*Itgam*), CD16 (*Fcgr3*), and CD32 (*Fcgr2*), which was mirrored by an upregulation of MR (*Mrc1*), Dectin-1 (*Clec7a*), DC-SIGN (*Cd209*), and CD36 (*Cd36*) alternative activation markers (Figure 1A). This finding was further supported by the reciprocal increase of anti-inflammatory IL-10 (*Il10*), IL-1 receptor antagonist (*Il1ra*), TGFβ (*Tgfb1*), CCL-17 (*Ccl17*), and CCR2 (*Ccr2*) and reduction of IL-12 (*Il12*) and CCL-2 (*Ccl2*) mRNA expression. Moreover, consistent with increased mRNA encoding alternative M2 activation markers, protein levels of MR, Dectin-1, and IL-10 were significantly increased (Figures 1B,C). The acquisition of this alternative phenotype upon P17 activation is reinforced by the strong decrease of IL-12 protein level (Figure 1C). We observed unaffected protein amounts for DC-SIGN, CD16, and CD36 (Figure 1B).

Surprisingly, the increase in alternative markers in P17-treated h-MDMs was accompanied by an inflammatory signature, as demonstrated by an augmentation in the mRNA level of the pro-inflammatory cytokines IL-1β (*Il1b*), TNF-α (*Tnf*α), and IL-6 (*Il6*). Consistently, the production of IL-1β and TNF-α was also induced in P17-treated h-MDMs (Figure 1C). In line with increased IL-1β production in P17-treated h-MDMs, the processing of pro-caspase-1 into its p20 subunit, which is a hallmark of caspase-1 activation, was significantly increased in P17-treated h-MDMs, demonstrating that the caspase-1-induced IL-1β release is activated by P17 (Figure 1D).

To further explore the pro-inflammatory impact of P17 on h-MDMs, we next assessed ROS production by P17-treated h-MDMs. We demonstrated that ROS release was strongly increased in P17-treated h-MDMs (Figure 1E). In line, the mRNA level and the amount of phosphorylated p47^phox^, a cytosolic subunit of the NADPH oxidase complex whose activation is essential to ROS release, were significantly increased in h-MDMs activated by P17 (Figures 1F,G).

Taken together, these results indicated that P17 induces an alternative phenotype characterized simultaneously by a CLRs signature composed of MR and Dectin-1 and a pro-inflammatory profile.

### P17 Induces MR and Dectin-1 Expression on h-MDMs through PPARγ Activation Dependent on LTB4 Production

The nuclear receptor PPARγ is a key component of the signaling pathway leading to alternative activation of macrophages and directly controls the expression of CLRs (34–36). To identify how P17 may have an impact on MR and Dectin-1 overexpression, we evaluated whether P17 can regulate PPARγ activation. The mRNA levels of PPARγ (*Pparg*) and of its target gene SRB1 (*Scarb1*) were significantly increased in P17-treated h-MDMs, suggesting that P17 improved PPARγ activation.

**Figure 2:**
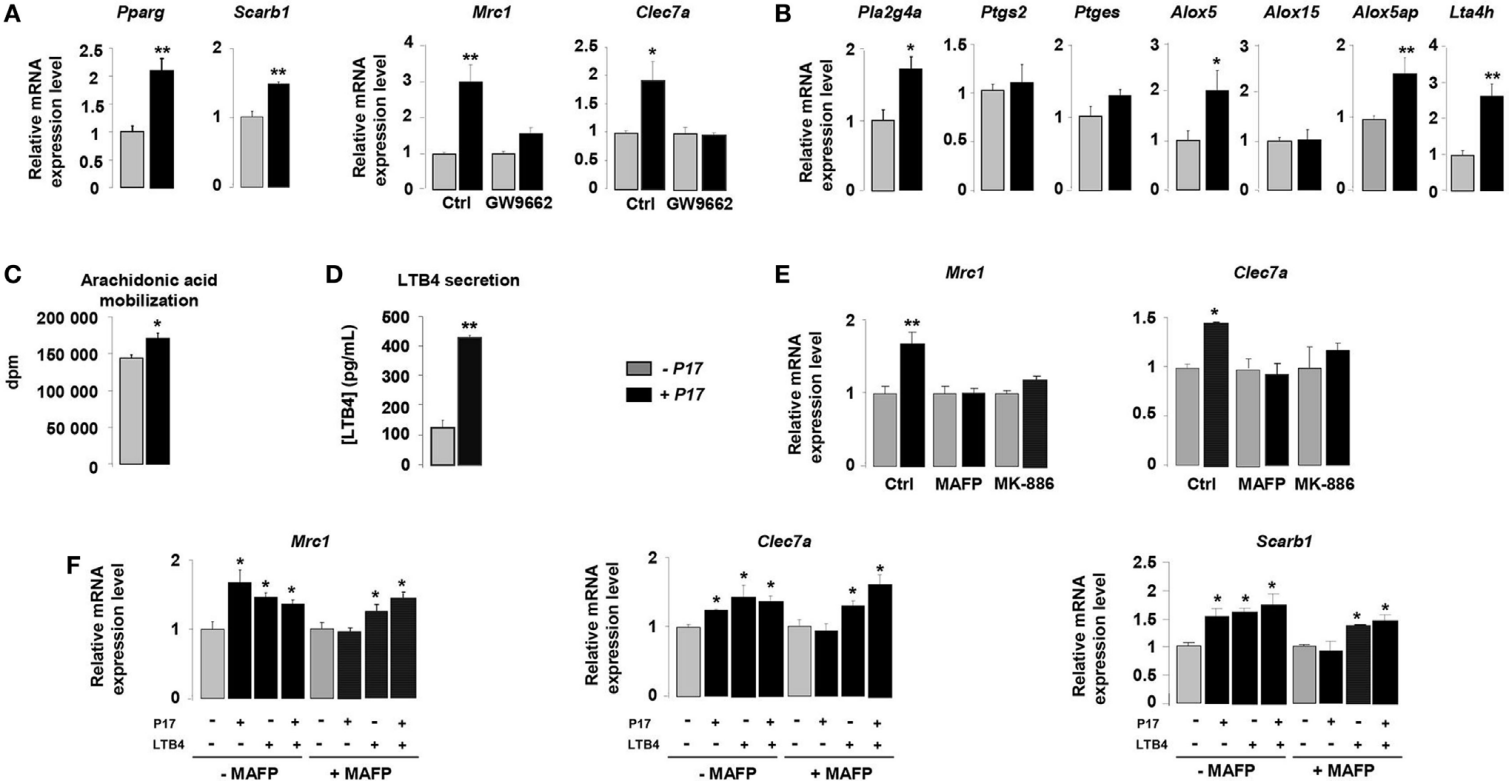
P17 promotes C-type lectin receptor (CLR) expression on human monocyte-derived macrophages (h-MDMs) through leukotriene B4 (LTB4)-mediated peroxisome proliferator-activated receptor gamma (PPARγ) activation. **(A)** Gene expression analysis of PPARγ, SRB1, mannose receptor (MR), and Dectin-1 in h-MDMs treated or not with P17, in the presence of a selective PPARγ antagonist GW9662. The results were represented in fold induction relative to the untreated h-MDMs control. **(B)** Gene expression analysis of arachidonic acid metabolic enzymes in h-MDMs treated or not with P17. The results were represented in fold induction relative to the untreated h-MDMs control. **(C)** [^3^H]AA mobilization in membrane phospholipids of h-MDMs treated or not with P17. **(D)** LTB4 production by h-MDMs treated or not with P17. **(E)** Gene expression analysis of MR and Dectin-1 in h-MDMs treated or not with P17, in the presence of MAFP, a specific cPLA2 inhibitor, and MK-886, a FLAP inhibitor, which prevents 5-LOX activation. The results were represented in fold induction relative to the untreated h-MDMs control. **(F)** Gene expression analysis of MR, Dectin-1, SRB1, and CD36 in h-MDMs treated or not with P17, in the presence of MAFP and/or LTB4. The results were represented in fold induction relative to the untreated h-MDMs control. Results correspond to mean ± SEM of triplicates. Data are representative of three independent experiments. *p < 0.05, **p < 0.01 compared to the respective untreated control.

To further explore whether P17-induced PPARγ activation was involved in MR and Dectin-1 overexpression, we evaluated the gene expression of these two CLRs in the presence of GW9662, a selective PPARγ antagonist. The treatment of P17-activated h-MDMs with GW9662 inhibited Mrc1 and Clec7a gene overexpressions, demonstrating that PPARγ is critically required for P17-induced Dectin-1 and MR expression on human h-MDMs (Figure 2A).

Peroxisome proliferator-activated receptor gamma is activated by endogenous ligands derived from AA (34, 35, 43, 48). The COX-1/2 cyclooxygenase, 5- and 12/15-LOX are considered to be critical for the conversion of AA into endogenous PPARγ ligands. To assess whether P17 can coordinate PPARγ ligand availability, we evaluated the gene expression of enzymes involved in both COX and LOX signaling pathways. The mRNA levels of cPLA2 (*Pla2g4a*), enzyme needed for AA release from membrane phospholipids, 5-LOX (*Alox5*), FLAP (5-LOX activating enzyme) (*Alox5ap*), and LTA4 hydrolase (*Lta4h*), critical for LTB4 synthesis, were strongly increased in P17-treated h-MDM (Figure 2B). Moreover, the mRNA levels of COX-2 (*Ptgs2*), PGES (*Ptges*), and 15-LOX (*Alox15*) were not differentially expressed in untreated and P17-treated h-MDMs, supporting that P17 had no incidence on COX-2 and 12/15-LOX signaling pathways.

Then, we determined whether these effects on gene expression also translate into changes in ligand availability. Consistent with cPLA2 gene overexpression, the mobilization of AA was induced in P17-treated h-MDMs (Figure 2C). Furthermore, in line with LTA4 hydrolase expression in P17-treated h-MDMs, we observed strong increase of LTB4 release (Figure 2D). These data suggest that the generation of LTB4 metabolites by h-MDMs upon P17 treatment is dependent both on AA mobilization and metabolism.

To confirm that P17 positively regulates MR and Dectin-1 expressions through the AA metabolism, we determined Mrc1 and Clec7a gene expression in the presence of MAFP, a specific cPLA2 inhibitor, and MK-886, a FLAP inhibitor, which prevents 5-LOX activation. The increase of MR and Dectin-1 mRNA levels in P17-treated h-MDMs was completely lost in the presence of MAFP and MK-886 (Figure 2E), further supporting that P17 controls MR and Dectin-1 expressions through AA metabolism.

Peroxisome proliferator-activated receptor gamma activity, as determinate by the induction of MR-, Dectin-1- and SRB1-specific PPARγ target genes, was increased similarly with P17 and LTB4 (Figure 2F). Furthermore, the addition of both P17 and LTB4 did not showed any additive effect, suggesting that P17 regulates MR and Dectin-1 surface expression by controlling PPARγ activation through the LTB4 production. Interestingly, the inhibition of P17-induced PPARγ target genes expression by MAFP was restored by the addition of LTB4, clearly establishing that P17-mediated PPARγ activation through LTB4 synthesis. Overall these data showed that P17 induces alternative activation of h-MDMs characterized by Dectin-1 and MR expressions through LTB4-mediated PPARγ activation.

### P17 Improves Fungicidal Properties of h-MDM through AA/LTB4/PPARγ/MR-Dectin-1 Signaling

**Figure 3:**
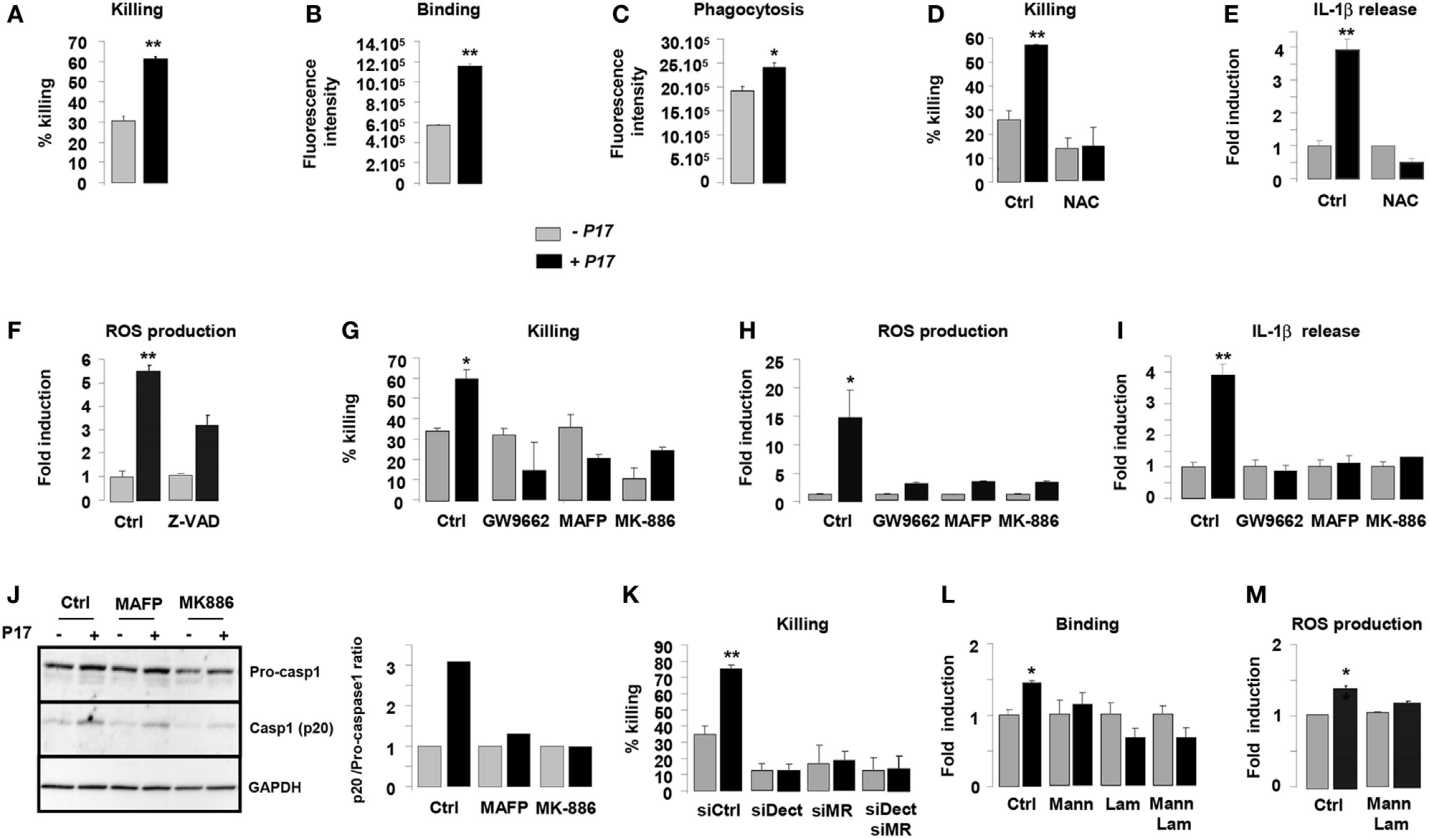
P17 improves anti-fungal properties of human monocyte-derived macrophages (h-MDMs) through AA/LTB4/peroxisome proliferator-activated receptor gamma (PPARγ)/Dectin-1-MR signaling pathway. **(A)** Killing assay of h-MDMs treated or not with P17 incubated with *Candida albicans*. Binding **(B)** and phagocytosis **(C)** of *C. albicans* by h-MDMs treated or not with P17. **(D)** Killing assay of h-MDMs treated or not with P17 incubated with *C. albicans* in presence of antioxidant *N*-acetyl cysteine (NAC). **(E)** Interleukin (IL)-1β release by h-MDMs treated or not with P17 after *C. albicans* challenge in presence of antioxidant *N*-acetyl cysteine (NAC). The results were represented in fold induction relative to the respective untreated h-MDMs control. **(F)** Reactive oxygen species (ROS) production by h-MDMs treated or not with P17 after *C. albicans* challenge in presence of caspases inhibitor ZVAD. The results were represented in fold induction relative to the respective untreated h-MDMs control. **(G)** Killing assay of h-MDMs treated or not with P17 incubated with *C. albicans* in presence of a selective PPARγ antagonist (GW9662), a specific inhibitor of arachidonic acid (AA) mobilization (MAFP), or of an inhibitor of 5-LOX activation (MK-886). ROS **(H)** and IL-1β **(I)** production by h-MDMs treated or not with P17 after *C. albicans* challenge in presence of a selective PPARγ antagonist (GW9662), a specific inhibitor of AA mobilization (MAFP), or of an inhibitor of 5-LOX activation (MK-886). The results were represented in fold induction relative to the respective untreated h-MDMs control. **(J)** Immunoblot analysis of caspase-1 p20 fragment cleavage in h-MDMs treated or not with P17 after *C. albicans* challenge in presence of a specific inhibitor of AA mobilization (MAFP) or of 5-LOX activation inhibitor (MK-886). Band intensity was quantified with Image J software and was represented as the ratio between the band intensities of p20 and of pro-caspase-1. **(K)** Killing assay of h-MDMs silenced for Dectin-1 and/or mannose receptor (MR) treated or not with P17 after *C. albicans* challenge. **(L)** Binding of *C. albicans* by h-MDMs treated or not with P17 in presence of mannan and/or laminarin. The results were represented in fold induction relative to the respective untreated h-MDMs control. **(M)** ROS production by h-MDMs silenced for Dectin-1 and MR treated or not with P17 after *C. albicans* challenge. The results were represented in fold induction relative to the respective untreated h-MDMs control. Results correspond to mean ± SEM of triplicates. Data are representative of three independent experiments. *p < 0.05, **p < 0.01 compared to the respective untreated control.

Previous works from our laboratory established the importance of alternative activation in the fungicidal functions of macrophages (34, 35, 43). On the basis of the current findings demonstrating an effect for P17 on alternative polarization, we next investigated whether P17-treated h-MDMs could have an impact on the *C. albicans* clearance. P17 did not exhibit an effective anti-fungal activity against *C. albicans* relative to the conventional amphotericin B anti-fungal agent. Furthermore, the association of P17 with amphotericin B did not improve the AMB anti-fungal activity, supporting that this peptide could not be used as a direct anti-fungal agent (Figure S1A in Supplementary Material). After P17 treatment, h-MDMs showed a robust increase in their ability to kill *C. albicans*, demonstrating that P17 promotes macrophage-intrinsic anti-fungal activity and supporting the use of P17 as a promising immunomodulatory compound to restrain fungal infections (Figure 3A). Consistent with our observation, P17-treated h-MDMs were more efficient to bind and to engulf *C. albicans* (Figures 3B,C). Interestingly, the induction of ROS production by P17 is essential in *in vitro* fungicidal activity of P17-activated h-MDMs, since the use of antioxidant *N*-acetyl cysteine (NAC) totally abolished the fungicidal effect of P17-treated h-MDMs against *C. albicans* (Figure 3D).

Then, we examined whether ROS production, which act as a common cellular signal upstream of the inflammasome activation (49), was responsible for IL-1β induction by P17 in response to *Candida*. While the antioxidant NAC suppressed *Candida*-induced IL-1β secretion by P17-activated h-MDMs, the inhibition of caspase activation by the addition of ZVAD did not change ROS production by P17-activated h-MDMs after *Candida* challenge (Figures 3E,F). Therefore, these results showed that in response to *Candida*, ROS production occurred upstream of the caspase-1-induced IL-1β production.

To determine whether P17-mediated PPARγ activation dependently on LTB4 synthesis was involved in fungicidal activity of P17-treated h-MDMs, we evaluated their ability to kill *C. albicans* in the presence of a selective PPARγ antagonist GW9662, a specific inhibitor of AA mobilization MAFP, or an inhibitor of 5-LOX activation MK-886. The increased capacity of P17-treated h-MDMs to kill *C. albicans* was totally inhibited in presence of GW9662, MAFP, and MK-886 (Figure 3G). Consistent with these findings, the addition of these inhibitors abolished P17-mediated ROS, IL-1β release, and caspase-1 activation of h-MDMs in response to *C. albicans* (Figures 3H–J).

To explore whether the increased expression of Dectin-1 and MR on P17-treated h-MDMs has any functional consequence in *C. albicans* elimination, we assessed the ability of P17-treated h-MDMs deficient for Dectin-1 and/or MR to kill the yeast. The gene silencing for Dectin-1 and/or MR in P17-treated h-MDMs resulted in the abolition of the ability of these cells to kill more efficiently *C. albicans* (Figure 3K). In terms of recognition of *C. albicans*, the pre-treatment of P17-activated h-MDMs with soluble MR and Dectin-1-blocking agents (mannan and/or laminarin, respectively) abrogated the increased capacity for P17-treated h-MDMs to interact with the yeast (Figure 3L). Moreover, this pre-treatment totally abolished the induction of ROS production after *C. albicans* challenge (Figure 3M).

Taken together, these data provided evidence that MR and dectin-1 are critical in fungicidal properties of h-MDMs mediated by P17 and support the importance of AA/LTB4/PPARγ/Dectin-1-MR axis in the strengthening of anti-fungal functions of h-MDMs by this HDP.

### The Interaction between P17 and GPCR Controls Anti-Fungal Properties of h-MDM *via* the Induction of Intracellular Calcium Mobilization

**Figure 4:**
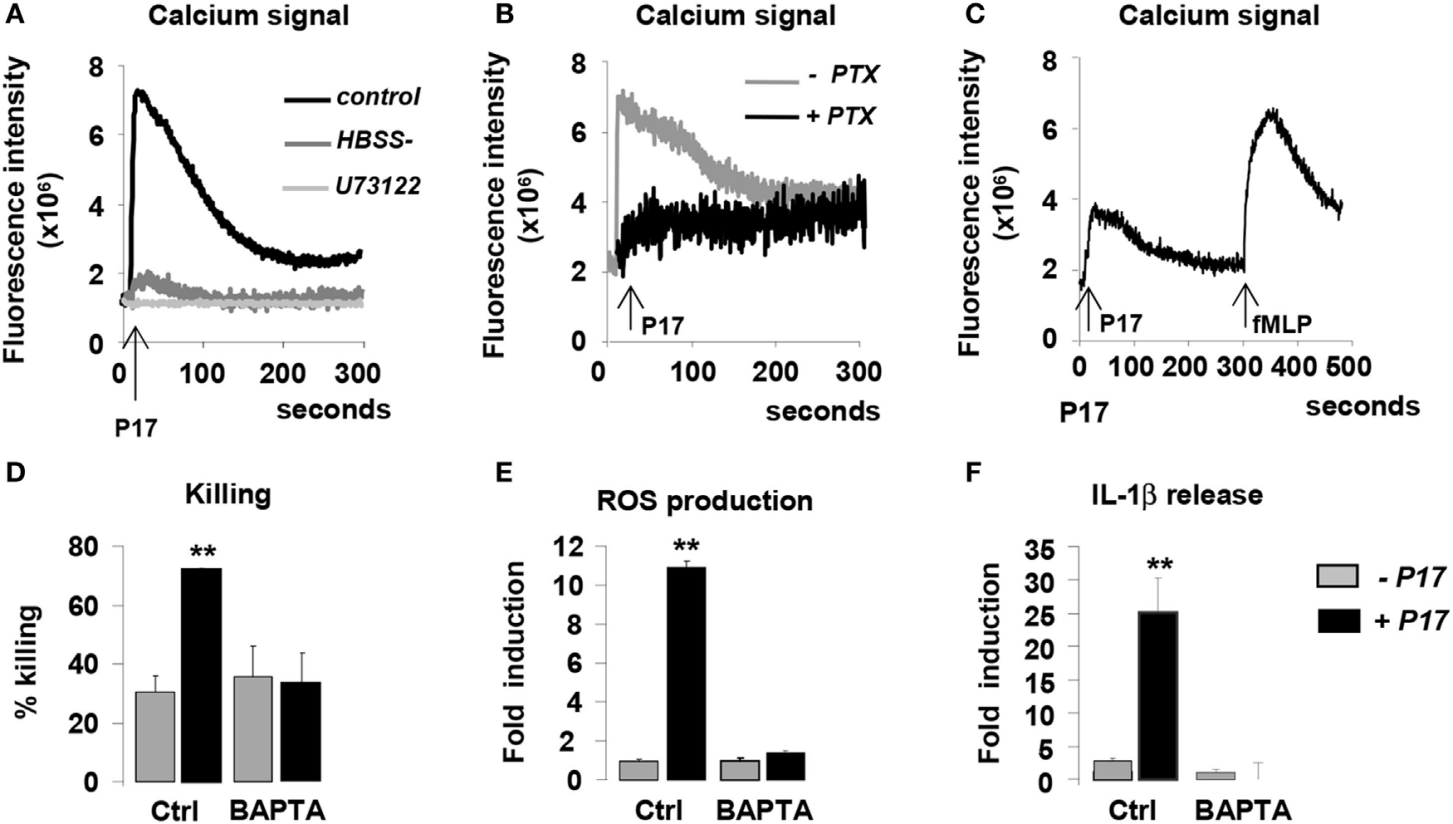
P17 controls anti-fungal properties of human monocyte-derived macrophages (h-MDMs) *via* the induction of intracellular calcium mobilization dependent on pertussis toxin (PTX)-sensitive G-protein-coupled receptor interaction. Intracellular calcium concentration in h-MDMs treated or not with P17 using fluorescent probe Fluo 3-AM. **(A)** Calcium concentration in h-MDMs treated or not with P17 in the presence of calcium-deprived medium [Hank’s balanced salt solution (HBSS-)] and an inhibitor of the intracellular pools calcium release (U73122). **(B)** Calcium concentration in h-MDM treated or not with P17 in the presence of PTX. **(C)** Calcium concentration in h-MDMs treated or not with P17 after desensitization of *N*-formylmethionine-leucyl-phenylalanine peptide receptors. **(D)** Killing assay of h-MDMs treated or not with P17 incubated with *Candida albicans* in the presence of calcium chelator (BAPTA-AM). Reactive oxygen species **(E)** and interleukin-1β **(F)** release by h-MDMs treated or not with P17 in the presence of calcium chelator (BAPTA-AM) after *C. albicans* challenge. The results were represented in fold induction relative to the respective untreated h-MDMs control. Results correspond to mean ± SEM of triplicates. Data are representative of three independent experiments. *p < 0.05, **p < 0.01 compared to the respective untreated control.

Several studies demonstrated that HDPs induce intracellular calcium mobilization in immune cells (50–53). On this basis, we evaluated cytosolic calcium concentration in P17-activated h-MDMs. P17 treatment induced a significant augmentation of intracellular calcium concentration in h-MDMs, indicating that this HDP triggers intracellular calcium signal (Figure 4A). In order to determine the source of this calcium release, we assessed calcium mobilization in calcium-deprived medium to inhibit extracellular calcium influx (HBSS) and in the presence of U73122, an inhibitor of the intracellular pools calcium release. Interestingly, calcium mobilization was abolished both in presence of HBSS and U73122 (Figure 4A), suggesting that the P17-increase intracellular calcium concentration in h-MDMs was dependent on extracellular calcium influx and intracellular calcium stores mobilization.

Most of the HDPs immunomodulatory functions are mediated through GPCRs (27–29, 51). In order to evaluate the involvement of these receptors in the activation of h-MDMs mediated by P17, we used pertussis toxin (PTX), known to inhibit some GPCRs. PTX treatment totally inhibited the mobilization of calcium in P17-activated h-MDMs (Figure 4B), suggesting that P17 activity on h-MDMs was mediated by PTX-sensitive GPCR interaction. Interestingly, although PTX-sensitive fMLP receptors 1 and 2 (FPR1 and FPR2) are the main GPCRs involved in the modulation of immune response by HDPs, we demonstrated here that the desensitization of these receptors by the addition of fMLP did not affect P17-induced calcium mobilization (Figure 4C).

To investigate the role of P17-induced calcium mobilization in the fungicidal properties of P17-treated h-MDMs, we evaluated their capacity to kill the yeasts in the presence of an intracellular calcium chelator BAPTA-AM. The increase of ability to kill *C. albicans* of P17-activated h-MDMs was completely lost in the presence of BAPTA-AM (Figure 4D). These findings were consistent with the lack of induction of ROS and IL-1β production in BAPTA-AM pretreated h-MDMs activated with P17 (Figures 4E,F), further supporting that the intracellular mobilization of calcium is essential in fungicidal properties of P17-treated h-MDMs. Altogether, these data highlight that P17 modulates anti-fungal immune response of h-MDMs through PTX-sensitive GPCR-triggered intracellular calcium mobilization.

### *In Vivo* P17 Treatment Induces Fungicidal Properties of Macrophages Hence Improving the Regression of Gastrointestinal Candidiasis

**Figure 5:**
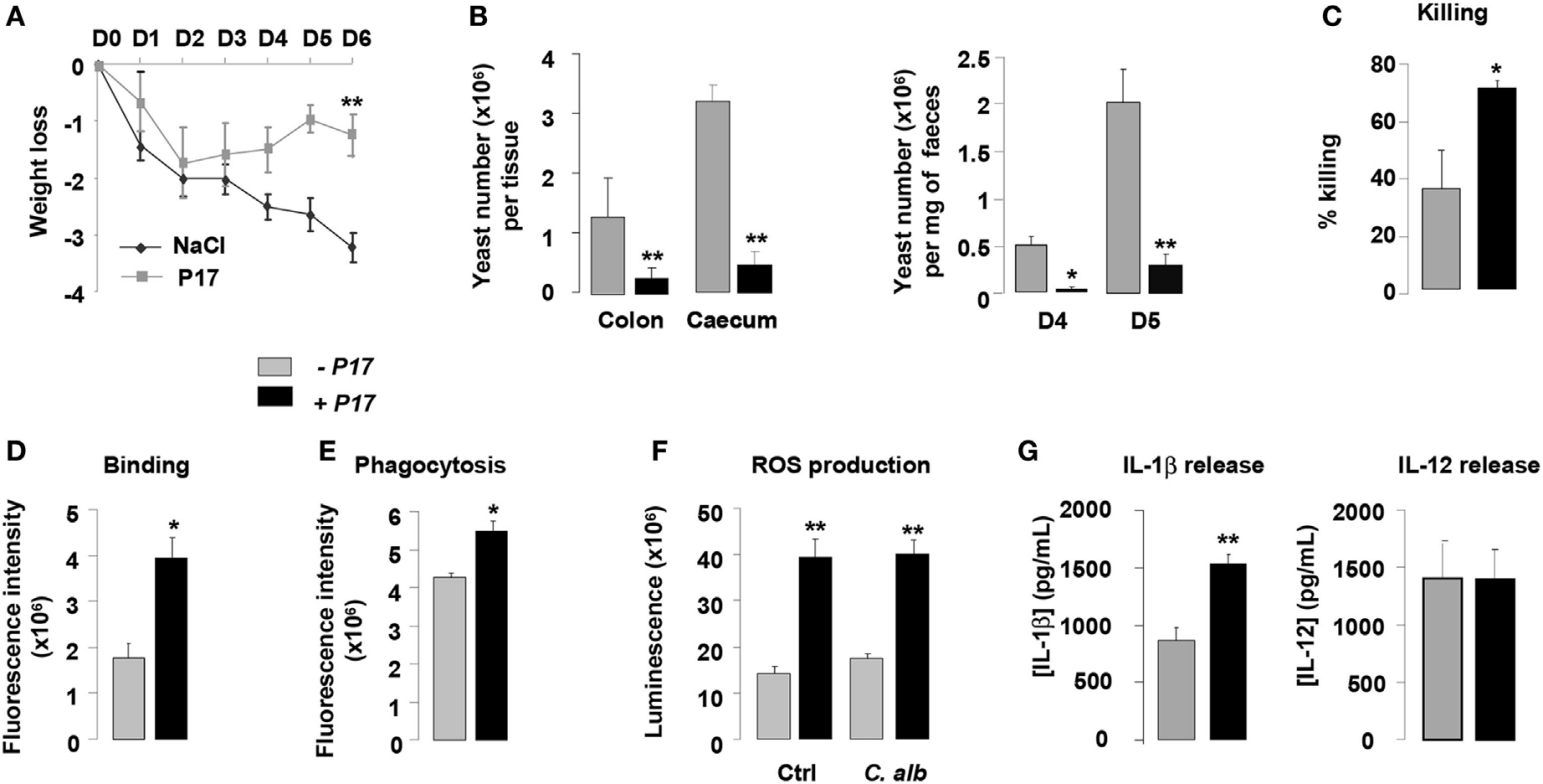
P17 treatment of mice improves macrophage-intrinsic anti-fungal activities and restrains gastrointestinal fungal infection. **(A)** Body weight of untreated or P17-treated mice during *Candida albicans* gastrointestinal infection. **(B)** *C. albicans* GI colonization in cecum and colon of untreated or P17-treated mice determined on day 6 post-infection. *C. albicans* GI colonization in feces of untreated or P17-treated mice determined on days 4 and 5 post-infection. **(C)** Killing assay of peritoneal macrophages from untreated or P17-treated infected mice. Binding **(D)** and Phagocytosis **(E)** of *C. albicans* by peritoneal macrophages from untreated or P17-treated infected mice. Reactive oxygen species (ROS) **(F)** and interleukin (IL)-1β and IL-12 **(G)** production upon *Candida* challenge of peritoneal macrophages from untreated or P17-treated infected mice. Results correspond to mean ± SEM of triplicates. Data are representative of at least two independent experiments (*n* = 6 per group). *p < 0.05, **p < 0.01 compared to the respective untreated control.

On the basis of the current findings demonstrating the microbicidal activity of P17-treated h-MDM against *C. albicans*, we next explored whether the P17 treatment could have an impact on the *in vivo* candidiasis outcome. In this context, we evaluated the fungal burden in the intestinal tract and the macrophage microbicidal functions in a murine experimental model of gastrointestinal candidiasis. P17-treated mice (P17) infected with *C. albicans* developed less severe gastrointestinal infection than untreated mice (NaCl), as demonstrated by lesser weight loss and by reduced fungal burden as reflected by a decreased number of CFU in the colon, cecum, and feces of mice treated with P17 (Figures 5A,B).

To investigate whether the effect of P17 on decreased *Candida* gastrointestinal colonization can be correlated to its impact on fungicidal functions of macrophages, we evaluated the capacity of macrophages from P17-treated mice to kill yeasts *in vitro*. Compared to macrophages from untreated mice, macrophages from P17-treated mice showed an increase in their ability to kill *C. albicans* (Figure 5C). Consistent with our observation, macrophages from P17-treated mice were more efficient in engulfing *C. albicans* and producing ROS and IL-1β (Figures 5D–G). Moreover, IL-12 release was similar in macrophages from untreated and P17-treated mice, demonstrating that P17 *in vivo* administration did not impact on IL-12 macrophage production (Figure 5G).

Taken together, these data provide *in vivo* evidence that P17 improves macrophage-intrinsic anti-fungal activity and support that P17 may constitute promising compound to restrain gastrointestinal fungal infection.

## Discussion

Although the majority of HDPs are currently known to exert antimicrobial activities against a broad spectrum of pathogenic microorganisms, they also can modulate the functions of immune cells (8). Among the two major groups of HDPs in humans, cathelicidins, and defensins, the LL-37 cathelicidin is the major AMP described for its immunomodulatory properties. Indeed, the LL-37 controls in several cells, particularly in human monocytes and murine macrophages, the transcription and the secretion of pro-inflammatory cytokines and chemokines (14–16, 18). Thus, the LL-37 participates to the immune cell differentiation, activation and chemotaxis (13, 54). Altogether these properties confer to LL-37 a strong microbicidal activity through its involvement in the control of inflammatory and anti-infectious signaling in macrophages. These immunomodulatory functions on human and murine cells are also described for HDPs from arthropods, such as apidaecin, bee venom AMP, and venom peptides 7.2 and 7.8, parabutoporin and opistoporin, isolated from scorpion venom (25, 26, 55). Our team has recently isolated a new short HDP from *T. bicarinatum* ant venom, called P17. Previous results demonstrated that this HDP shares common structural properties with LL-37. Indeed, structural studies of P17 revealed that this ant HDP, composed of 13 amino acids, is cationic, amphipatic, amidated in C-terminal position, and adopts an alpha helix conformation (31).

Here, we reported that the P17 influences phenotypic differentiation of h-MDMs toward an alternative phenotype characterized by a CLRs signature composed of MR and Dectin-1. Consistent with the key role of MR and Dectin-1 in yeast recognition, phagocytosis and clearance (35, 38, 56, 57), we demonstrated that the P17 increases the ability of h-MDMs to eliminate *C. albicans*. The involvement of MR and Dectin-1 in the P17-mediated anti-fungal activity of h-MDMs was further evidenced by the loss of *Candida* elimination in h-MDMs silenced for MR or Dectin-1. In line, the pre-treatment of P17-activated h-MDMs with soluble MR or Dectin-1 blocking agents, also severely compromised their capacity to bind and to kill *C. albicans*. Consistent with the cooperative role of MR and Dectin-1 in the induction of macrophage anti-fungal signaling pathways (34, 35, 37), we demonstrated that both MR and Dectin-1 are involved in the recognition of this yeast and in its subsequent elimination by P17-activated h-MDMs. Consistently, we showed that P17-activated h-MDMs is able to clear *Leishmania infantum* (Figures S1B,C in Supplementary Material), a parasite known to interact with macrophages through MR and Dectin-1 (36). These results reveal that the immunomodulatory activity of P17 presents a broader spectrum, as long as the pathogen expresses on its surface carbohydrates recognized by MR or Dectin-1.

This study also provided the mechanistic insight into the transcriptional control of MR and Dectin-1 by P17 in h-MDMs. On the basis of the established role of PPARγ in the alternative activation and in the control of CLRs expression (34, 58), these findings identify PPARγ as a critical component in the signaling cascade that drives P17-mediated MR and Dectin-1 overexpression.

In addition to the transcriptional increase of MR and Dectin-1 in h-MDM by P17 treatment, we demonstrated that this HDP positively regulates the transcription of cPLA2, enzyme needed for AA release from membrane phospholipids, of 5-LOX, FLAP, and LTA4 hydrolase, critical for LTB4 synthesis. Consistent with this observation, the mobilization of AA and the generation of LTB4 by h-MDMs upon P17 treatment are strongly increased. Interestingly, the impairment of LTB4 production by a specific inhibitor of 5-LOX activation, completely abolishes the induction of MR and Dectin-1 mediated by P17, suggesting that P17 controls MR and Dectin-1 expressions through the LTB4 release. Moreover, the fact that P17 increases MR-, Dectin-1-, and SRB1-specific PPARγ target genes, similar to LTB4, and that the addition of both P17 and LTB4 did not show any additive effect on these gene inductions supports that P17 regulates MR and Dectin-1 surface expressions by controlling PPARγ activation through the LTB4 production. This is reinforced by the finding showing that the inhibition of P17-induced MR, Dectin-1, and SRB1 PPARγ target gene expressions by MAFP, a specific inhibitor of AA release, was restored by the addition of LTB4. In agreement with the identification of LTB4 as PPARγ agonist in P17-mediated CLRs induction, numerous endogenous PPARγ ligands derived from the metabolism of AA are described (34, 35, 43, 48).

A significant contribution of this study was the identification of a novel signaling pathway triggered by an HDP involved in the antimicrobial response of macrophages. Indeed, the h-MDMs treated with PPARγ-specific antagonist, or with inhibitors of LTB4 synthesis, or silenced for MR or Dectin-1 failed to increase the killing of *C. albicans* in response to P17, establishing LTB4/PPARγ/Dectin-1-MR axis as crucial in the acquisition of anti-fungal properties of P17-treated h-MDMs.

Because ROS and pro-inflammatory cytokines are essentials *Candida*-killing components (35, 37, 39, 59, 60), we evaluated ROS and IL-1β release of h-MDMs upon P17 treatment and we investigated the signaling pathways involved in their production. Remarkably, the alternative phenotype of P17-treated h-MDMs is accompanied by an inflammatory signature characterized by IL-1β and ROS productions. In line, previous studies have highlighted the capacity of HDPs to simultaneously promote pro-inflammatory response while protecting host organism against exacerbated inflammatory response (18, 25, 26, 61–64). Consistent with the increased IL-1β production in P17-treated h-MDMs, the processing of pro-caspase-1 into its p20 subunit, which is a hallmark of caspase-1 activation (65), is augmented in P17-treated h-MDMs. Moreover, the involvement of P17 in ROS production is supported by large amounts of phosphorylated p47^phox^, a cytosolic subunit of the NADPH oxidase complex whose activation is essential to ROS release (66), in h-MDMs activated by P17. We also established that this LTB4/PPARγ/Dectin-1-MR signaling drives ROS release in P17-treated h-MDMs, since the addition of GW9662, MAFP, and MK-886 or the pre-treatment of P17-activated h-MDMs with soluble MR- and Dectin-1-blocking agents abolishes P17-mediated ROS production by h-MDMs in response to *C. albicans*. This is supported by previous reports identifying Syk-dependent ROS production *via* Dectin-1 and MR receptors in fungal infection (67). Furthermore, we provided evidence for the major contribution of ROS production in IL-1β secretion by P17-activated h-MDMs in response to *Candida*. Consistent with these results, ROS production activates IL-1β processing *via* caspase-1-dependent activity (49). Thus, we identified both MR and Dectin-1 as extracellular sensors for *Candida* recognition by P17-activated h-MDMs and the subsequent activation of the pro-IL-1β synthesis and ROS production. These oxidant agents are essential in inflammasome-dependent IL-1β processing and hence in IL-1β production by P17-activated h-MDMs in response to *Candida* challenge. This is best supported by the fact that the induction of the Nlrp3 inflammasome complex activation is subjected to several events, such as the efflux of cellular potassium, the phagocytosis of particles, the generation of ROS, cathepsin B activation, and/or the vacuolar acidification (68–71).

Although we demonstrated with certitude that AA/LTB4/PPARγ/CLRs/ROS-IL-1β signaling pathway is involved in the anti-fungal activity of P17-activated h-MDMs, we also showed that P17 increases significantly the mRNA level of LL-37 (data not shown), the only human cathelicidin-related AMP known to promote a pro-inflammatory response (15, 16). Thus, LL-37, in addition to ROS and IL-1β, could be an important component of the anti-fungal activity of P17-activated macrophages. Interestingly, several studies show that PPARγ agonists promote the induction of cationic AMP expression (72). These observations support that the AA/LTB4/PPARγ axis triggered by P17 could also be involved in the production of LL-37.

One main implication of our study was the validation *in vivo* of the ability of P17 to modulate fungicidal activity of macrophages on a murine model of gastrointestinal candidiasis. The P17 treatment rendered the mice less susceptible to gastrointestinal *C. albicans* infection by promoting macrophage-intrinsic anti-fungal activity. These data reveal the P17 as an effective immunomodulatory agent for anti-fungal functions of macrophages.

Finally, this study identified that the interaction between P17 and GPCR is crucial in the induction of anti-fungal properties of h-MDMs by P17. Consistently, most HDPs control immune cells responses in a GPCR-dependent manner, mainly through fMLP receptors, such as FPR1 and FPR2 (27–29, 51). Although we demonstrated that P17 did not interact with fMLP receptors, this HDP engages PTX-sensitive GPCR, which is currently under identification. Moreover, in agreement with the involvement of calcium release in immunomodulatory activity triggered by many HDPs (50–53, 73), P17 modulates anti-fungal immune response of h-MDMs through PTX-sensitive GPCR-triggered intracellular calcium mobilization. As calcium signaling is an essential factor of cPLA2 activation in monocytes and macrophages (74, 75), P17 could promote LTB4/PPARγ/Dectin-1-MR signaling pathway through the activation of cPLA2 and the subsequent AA release.

**Figure 6:**
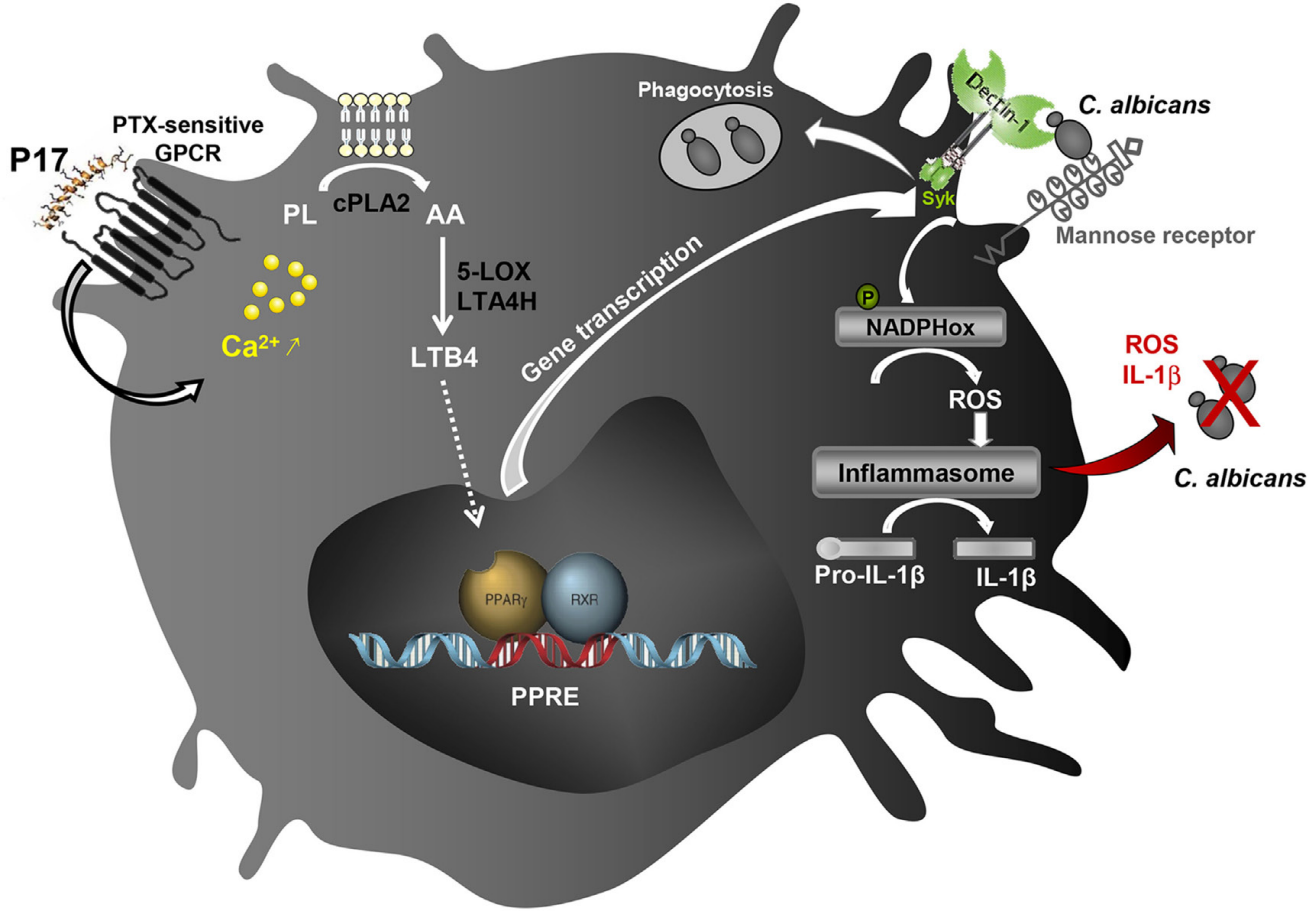
Schematic illustration of the immunomodulatory activity of P17 on human macrophages. The identification of molecular mechanisms triggered by P17 reveals that this peptide modulates anti-fungal immune response of human monocyte-derived macrophages (h-MDMs) through pertussis toxin (PTX)-sensitive G-protein-coupled receptor (GPCR)-triggered intracellular calcium mobilization. The increased calcium mobilization in P17-activated h-MDMs is essential in cPLA2 activation and the subsequent arachidonic acid (AA) release. This AA mobilization and the promotion of AA metabolization to leukotriene B4 (LTB4) upon P17 treatment are responsible of the peroxisome proliferator-activated receptor gamma (PPARγ)-dependent mannose receptor and Dectin-1 overexpression. The activation of AA/leukotriene B4 (LTB4)/PPARγ/Dectin-1–mannose receptor axis by P17 triggers reactive oxygen species (ROS) production and inflammasome-dependent interleukin (IL)-1β release critical in fungicidal activity of P17-activated h-MDMs.

Taken together, all these data highlighted the immunomodulatory activity of P17 on h-MDMs differentiation and their associated anti-fungal response. The identification of molecular mechanisms triggered by P17 responsible of increased microbicidal response of h-MDMs against *C. albicans* revealed the importance of the AA/LTB4/PPARγ/Dectin-1-MR axis (Figure 6). Confronted to the emergence of many resistances to the usual anti-infectious agents, P17 could constitute a promising compound to fight against fungal infections. Thus, this work offers new therapeutic perspectives and supports the use of PAMs as immunomodulatory compounds to restrain infectious diseases.

## Ethics Statement

Mononuclear cells were obtained from healthy blood donors (Etablissement Français du Sang, EFS Toulouse, France). Written informed consents were obtained from the donors under EFS contract no 21/PLER/TOU/UPS4/2013–0106. All mice were bred and handled by the protocols approved by the Conseil Scientifique du Centre de Formation et de Recherche Experimental Medico Chirurgical and the ethics board of the Midi-Pyrénées ethic committee for animal experimentation (Approval no B3155503).

## Author Contributions

AC, KB, MT, and BP designed the study and analyzed the data. AC and KB wrote the manuscript. KB, HA, and MP did and analyzed experiments. MA, LL, and MR did research. JL, AA and EB for helpful discussions.

## Conflict of Interest Statement

The authors declare that the research was conducted in the absence of any commercial or financial relationships that could be construed as a potential conflict of interest.
