# Supplementary figures and images for: P17, an Original Host Defense Peptide from Ant Venom, Promotes Antifungal Activities of Macrophages through the Induction of C-Type Lectin Receptors Dependent on LTB4-Mediated PPARγ Activation
Source: Front Immunol. 2017 Nov 30;8:1650. doi: 10.3389/fimmu.2017.01650 (PMC5716351; doi:10.3389/fimmu.2017.01650)

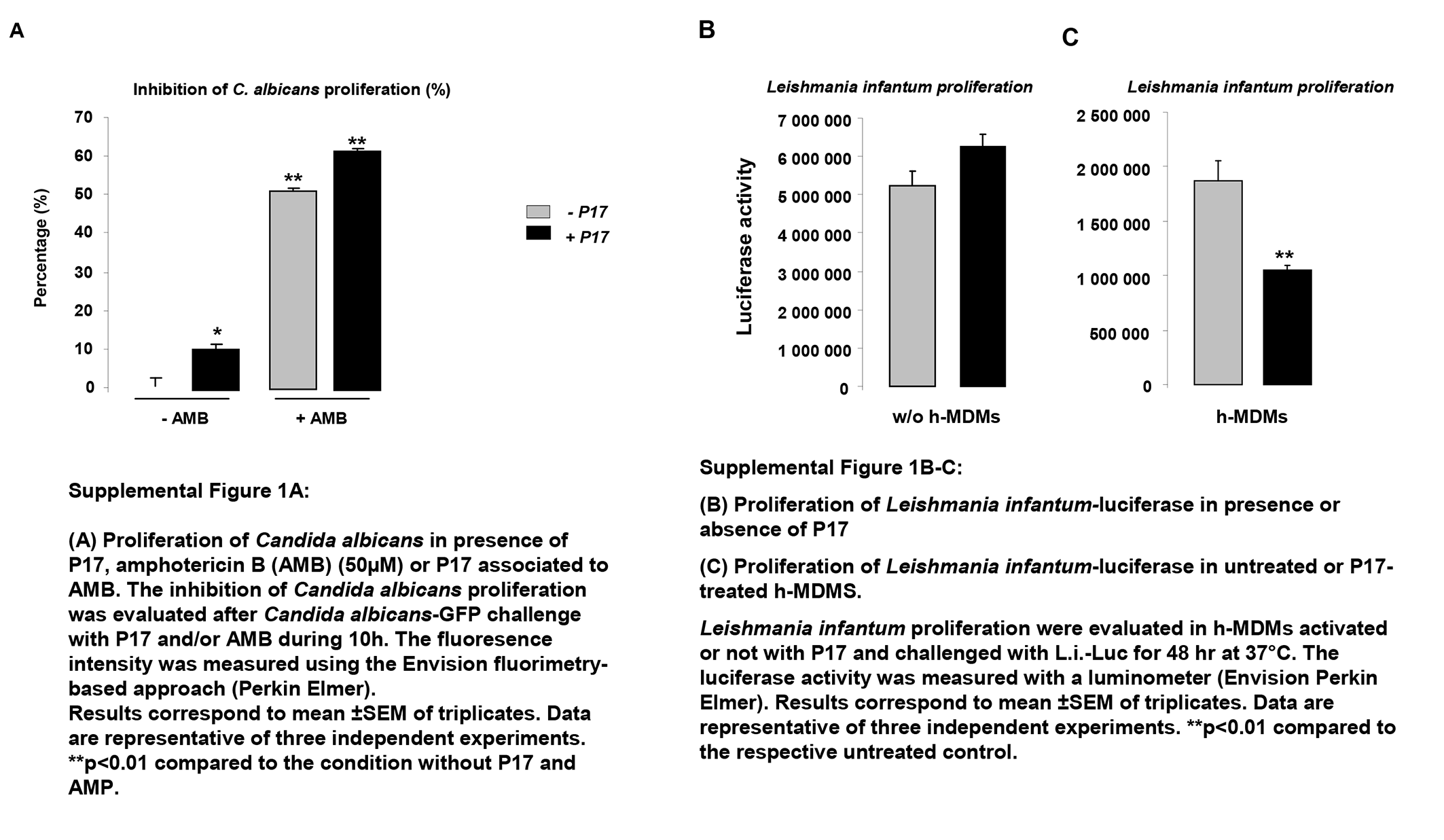

Supplement: Supplementary file 1 [file image_1.tif]
